# ﻿A new mountain pitviper of the genus *Ovophis* Burger in Hoge & Romano-Hoge, 1981 (Serpentes, Viperidae) from Yunnan, China

**DOI:** 10.3897/zookeys.1203.119218

**Published:** 2024-05-30

**Authors:** Xian-Chun Qiu, Jin-Ze Wang, Zu-Yao Xia, Zhong-Wen Jiang, Yan Zeng, Nan Wang, Pi-Peng Li, Jing-Song Shi

**Affiliations:** 1 Institute of Herpetology, Shenyang Normal University, Shenyang, Liaoning 110034, China Shenyang Normal University Shenyang China; 2 Department of Evolution, Ecology & Biodiversity, University of California, Davis, CA 95616, USA University of California Davis United States of America; 3 Institute of Zoology, Chinese Academy of Sciences, Beijing 100101, China Beijing Forestry University Beijing China; 4 School of Ecology and Nature Conservation, Beijing Forestry University, Beijing 100083, China Institute of Zoology, Chinese Academy of Sciences Beijing China; 5 Institute of Vertebrate Paleontology and Paleoanthropology, Chinese Academy of Sciences, Beijing 100044, China Institute of Vertebrate Paleontology and Paleoanthropology, Chinese Academy of Sciences Beijing China

**Keywords:** Morphology, *Ovophisjenkinsi* sp. nov., snake, taxonomy, Yingjiang County

## Abstract

Based on a molecular phylogenetic analysis and morphological comparison, a new species of mountain pitviper, *Ovophisjenkinsi***sp. nov.**, is described. The new species was collected in Yingjiang County, Yunnan Province, China. It can be distinguished from congeneric species by the following characters: (1) internasals in contact or separated by one small scale; (2) second supralabial entire and bordering the loreal pit; (3) dorsal scales in 23 (25)–21 (23, 25)–19 (17, 21) rows; (4) 134–142 ventrals; (5) 40–52 pairs of subcaudals; (6) third supralabial larger than fourth in all examined specimens of *Ovophisjenkinsi***sp. nov.**; (7) deep orange-brown or dark brownish-grey markings on dorsal head surface; (8) background color of dorsal surface deep orange-brown or dark brownish-grey; (9) both sides of dorsum display dark brown trapezoidal patches; (10) scattered small white spots on dorsal surface of tail.

## ﻿Introduction

The subfamily Crotalinae (pitvipers) is the largest group of family Viperidae, with 294 species in 23 genera, and widely distributed in Asia and America (Speybroeck et al. 2016; Uetz et al. 2024). The mountain pitviper (*Ovophis*) is a group of medium-sized venomous snakes that are mainly distributed through eastern Asia, the southern Himalayas, and the northern Indochina Peninsula (Che et al. 2020). Within the genus, the distribution of *O.monticola* (Günther, 1864), *O.makazayazaya* (Takahashi, 1922), *O.tonkinensis* (Bourret, 1934), and *O.zayuensis* (Jiang, 1977) in China was supported and defined preliminarily by Malhotra et al. (2011) by molecular phylogenetic analyses (12S, 16S, cytb, and ND4) and comparative morphology. Subsequently, Zeng et al. (2023) revised the molecular phylogeny (cytb, ND4, BACH1, c-mos, NT3, and Rag1) with additional specimens. The result restricted the distribution of *O.monticola* to Zhangmu County (southern Xizang) in China, demarcated the populations that are distributed through Sichuan–Yunnan in the west to Taiwan in the east as *O.makazayazaya*, and introduced *O.malhotrae*Zeng et al., 2023 as a new species representing the southern Yunnan population with its holotype description presented in non-paginationed supporting documents. The distribution of *O.malhotrae* is currently recorded only in Jinping and Pingbian, Yunnan and Lao Cai, Vietnam. MtDNA phylogenetic inference of the genus *Ovophis* and partial species of the rest of family Viperidae indicated when “*O.*” *okinavensis* (Boulenger, 1892) is included, genus *Ovophis* is polyphyletic, while “*O.*” *okinavensis* sistering *Trimeresurusgracilis* Oshima, 1920 (Malhotra and Thorpe 2000, 2004; Shi et al. 2021). Although the epithet change of “*O.*” *okinavensis* has not yet been declared, this species is no longer included in genus *Ovophis* in some recent taxonomic studies (Malhotra et al. 2011; Zeng et al. 2023). Currently, therefore, the genus *Ovophis* includes six species: *O.monticola*, *O.convictus* (Stoliczka, 1870), *O.makazayazaya*, *O.tonkinensis*, *O.zayuensis*, and *O.malhotrae* (Malhotra et al. 2011; Zeng et al. 2023; Uetz et al. 2024).

In 2018 and 2023, five specimens of genus *Ovophis* were collected in Yingjiang County, Yunnan. With applied comparative morphology and molecular phylogenetic analysis, these specimens were revealed as distinct from the other *Ovophis* species. Thus, we described here this new population as a new species.

## ﻿Materials and methods

### ﻿Sampling

Five specimens (IOZ 002679, IOZ 002680, YJ201801, YJ201802, and YJ201803) were collected by Zhong-Wen Jiang and Xian-Chun Qiu in October 2018 and 2023 from Tongbiguan Township, Yingjiang County, Yunnan Province, China. After euthanasia, liver tissues of specimens IOZ 002679 and IOZ 002680 were extracted and preserved in 95% ethanol for molecular analyses. All specimens were fixed in 10% buffered formalin and then transferred to 75% ethanol for permanent preservation. The specimens IOZ 002679 and IOZ 002680 are deposited in the Institute of Zoology, Chinese Academy of Sciences (**IOZ**, Beijing, China). The specimens YJ201801, YJ201802, and YJ201803 are deposited in Beijing Forestry University (**BFU**, Beijing, China).

### ﻿Morphometrics

Morphological descriptions are accorded to Zhao (2006). Abbreviations are accorded to Darko et al. (2022). A total of 24 morphological characters were examined, including 11 mensural characters and 13 scalation characters. Morphological measurements were taken with digital calipers (Guanglu 111N-101V, accuracy 0.03 mm, Guanglu Digital Instruments, Guilin) to the nearest 0.1 mm. Abbreviations are as follows: **SVL** snout–vent length (distance from tip of snout to posterior margin of cloacal plate); **TAL** tail length (distance from posterior margin of cloacal plate to tip of tail); **TL** total length (distance from tip of snout to tip of tail); **HL** head length (distance from tip of rostral to posterior end of jaw); **HW** head width (maximum width of head); **HH** head height (maximum height between dorsal and ventral surfaces of head); **ED** eye diameter (horizontal eye diameter); **IOD** interorbital distance (distance between the top margin of eyes); **IN** internarial distance (distance between nostrils); **RH** maximum rostral height; **RW** maximum rostral width; **LOR** loreal; **PRO** preoculars; **PO** postoculars; **SBO** suboculars; **ATEM** anterior temporals; **PTMP** posterior temporals; **SL** supralabials; **IL** infralabials; **CS** chin shields; **DSR** dorsal scale rows (counted at one head length behind head, at midbody, and one head length before vent); **PRV** preventral scales (elongated scales situtated beneath the head before the ventrals); **VS** ventral scales (elongated scales situtated beneath the body between neck and vent); **SC** subcaudal scales.

Other morphological characters of *Ovophis* species were obtained from Zhao et al. (1998), Li et al. (2010), Neang et al. (2011), Sharma et al. (2013), Che et al. (2020), Guo et al. (2021), Huang (2021), and Zeng et al. (2023).

### ﻿Phylogenetic analyses

Four mtDNA sequences are specifically amplified in this study: 12S rRNA using primers 12SFPhe and 12SRVal (Knight and Mindell 1993); 16S rRNA using primers 16sFL and 16sRH (Palumbi et al. 1991); cytochrome b (cytb) using primers L14910 and H16064 (Burbrink et al. 2000); and NADH dehydrogenase subunit 4 (ND4) using primers ND4F and LEUR (Arévalo et al. 1994). The standard PCR protocol is performed in 20 μl of reactant with at least 20 ng of template DNA and 10 pmol of primers. The PCR conditions: initial denaturation for 3 min at 94 °C, followed by 35 cycles, denaturation at 94 °C for 30 s, 30 s of annealing at different temperatures (52 °C for 12S, 50 °C for 16S, 56 °C for ND4, and 48 °C for cytb), and then elongation at 72 °C for 60 s, then finalized with elongation step of 10 min at 72 °C. Sequencing was conducted by Beijing Tianyi Huiyuan Bio-tech Co., Ltd. Sequence data were uploaded to GenBank (Table 1).

**Table 1.** Samples and sequences used for phylogenetic analysis in this study.

Species	Locality	Voucher	GenBank accession number			
			12S	16S	cytb	ND4
*Ovophisjenkinsi* sp. nov.	Yingjiang, Yunnan, China	IOZ 002679	PP574250	PP574252	PP171456	PP171459
*O.jenkinsi* sp. nov.	Yingjiang, Yunnan, China	IOZ 002680	PP574249	PP574251	PP171455	PP171458
*O.monticola*	Gandaki, Nepal	ZMB 70216	HQ325260	HQ325078	HQ325138	HQ325199
*O.monticola*	Gandaki, Nepal	ZMB 70218	HQ325253	HQ325071	HQ325131	HQ325192
*O.convictus*	Cameron Highlands, Pahang, Malaysia	AM B628	HQ325264	HQ325082	HQ325141	–
*O.convictus*	Pulau Langkawi, Malaysia	AM B629	HQ325265	HQ325083	HQ325142	–
*O.convictus*	Cameron Highlands, Pahang, Malaysia	AM B580	–	–	HQ325129	HQ325190
*O.malhotrae*	Yunnan, China	GP 2041	–	–	OP441841	OP441784
*O.malhotrae*	Jinping, Yunnan, China	GP 2053	–	–	OP441842	OP441785
*O.malhotrae*	Lao Cai, Vietnam	ROM 39381	HQ325283	HQ325102	HQ325160	HQ325218
*O.zayuensis*	Bomi, Xizang, China	GP 713	–	–	OP441890	OP441833
*O.zayuensis*	Chayu, Xizang, China	GP 1505	–	–	OP441892	OP441836
*O.makazayazaya*	Huili, Sichuan, China	GP 21	–	–	OP441856	OP441798
*O.makazayazaya*	Luquan, Yunnan, China	KIZ 02143	–	–	OP441860	OP441802
*O.makazayazaya*	Weixi, Yunnan, China	YPX 53011	–	–	OP441861	OP441803
*O.tonkinensis*	Maoming, Guangdong, China	GP 1665	–	–	OP441876	OP441818
*O.tonkinensis*	Xuan Son, Phu Tho, Vietnam	KIZ 011602	–	–	OP441880	OP441822
*Viperaberus*	Jilin, China	–	–	–	MF945570	MF945570

Note: The missing data are marked as “–”.

Corresponding homologous sequences of *Ovophis* species were obtained from GenBank, and the sequences of *Viperaberus* (Linnaeus, 1758) were used as outgroup in the phylogenetic analysis (Zeng et al. 2023). DNA nucleotide sequences were aligned in MEGA 6 (Tamura et al. 2013) with Clustal W algorithm, default parameters (Thompson et al. 1997). PartitionFinder 2.1.1 (Lanfear et al. 2012) was used to test the best partitioning scheme. Pairwise sequence divergence (uncorrected *p*-distances) was calculated using MEGA 6.

Bayesian inference was performed using MrBayes 3.1.2 (Ronquist et al. 2011). All searches consisted of three heated chains and a single cold chain. Three independent iterations each comprising two runs of 100 million generations, sampled every 10,000 generations, and parameter estimates were plotted against generation. The first 25% of the samples were discarded as burn-in, resulting in a potential scale reduction factor (PSRF) of < 0.005. A maximum-likelihood analysis was run with RaxML (Silvestro and Michalak 2012), and a majority rule consensus tree was calculated with 1,000 bootstrap replicates.

## ﻿Results

### ﻿Molecular phylogeny

**Figure 1.**
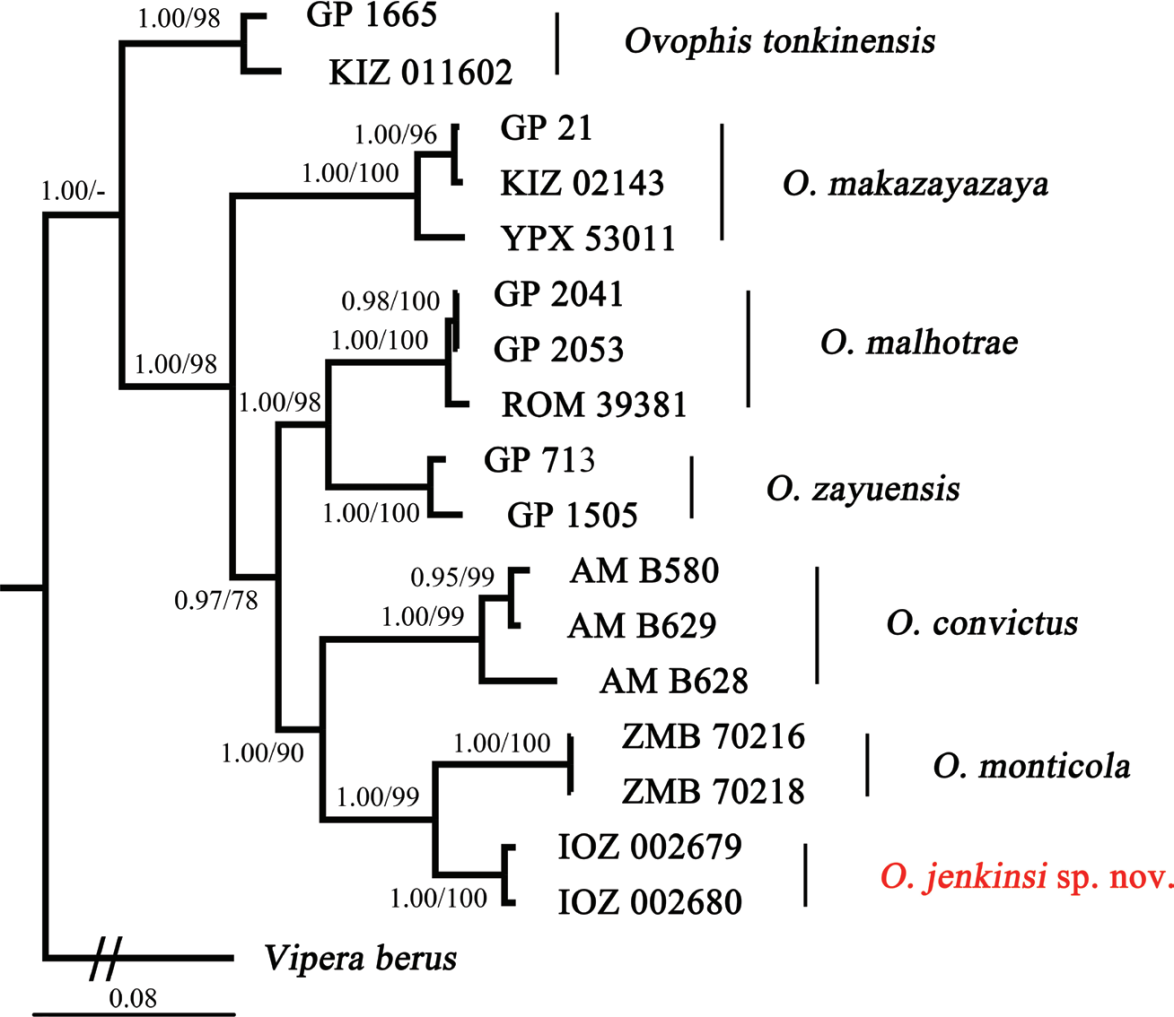
Bayesian-inference tree of *Ovophis* species inferred from the combined fragments of 12S, 16S, cytb, and ND4. Bayesian posterior probabilities/bootstrap support values for the clades are shown adjacent to the node.

A total of 2,703 aligned base pairs were obtained including 441 bp from 12S, 465 bp from 16S, 1,110 bp from cytb, and 687 bp from ND4. With respect to the different evolutionary characters of each molecular marker, the dataset was split into five partitions by genes and codon positions as recommended by PartitionFinder 2.1.1 (Table 2). The topological structures of the maximum-likelihood (ML) and Bayesian-inference (BI) trees are generally consistent (Fig. 1). All *Ovophis* samples are divided into seven clades. The cladistic relationship within the group of samples from Yingjiang was resolved with strong support (1.00/100 for BI and ML). The Yingjiang group is sister to the *O.monticola* group with decent support (1.00/99 for BI and ML), and the group of “Yingjiang”+*O.monticola* clustered with *O.convictus*, and formed a larger clade sister to the *O.malhotrae*+*O.zayuensis* lineage.

**Table 2.** Partitions and their molecular evolution models selected by PartitionFinder 2.1.1.

Partitions	Locus	Length (bp)	Models
Partition 1	12S, cytb pos 1, ND4 pos 3	1,040	TVM+I+G
Partition 2	16S	465	GTR+I
Partition 3	ND4 pos 1, cytb pos 2	599	TRN+I
Partition 4	cytb pos 3	370	TIM+G
Partition 5	ND4 pos 2	229	TIM+G

TVM: transversional substitution model; GTR: General Time-Reversible model; TRN: Tamura-Nei; TIM: transitional substitution model; +I: proportion of invariable sites; +G: rate heterogeneity.

The uncorrected *p*-distance based on cytb gene between the specimens IOZ 002679, IOZ 002680 from Yingjiang, Yunnan and *O.monticola* is 6.2–6.5%, equivalent to those among other recognized species, such as *O.malhotrae* vs. *O.zayuensis* (6.0–6.7%) (Table 3). Thus, the molecular phylogeny supports the validity of *Ovophisjenkinsi* sp. nov.

**Table 3.** Uncorrected *p*-distance among the sequences based on the cytb gene fragments of *Ovophis* species in this study.

	1	2	3	4	5	6	7	8	9	10	11	12	13	14	15	16
1 *Ovophisjenkinsi* sp. nov. IOZ 002679																
2 *O.jenkinsi* sp. nov. IOZ 002680	0.003															
3 *O.monticola* ZMB 70216	0.065	0.062														
4 *O.monticola* ZMB 70218	0.065	0.062	0.000													
5 *O.convictus* B580	0.097	0.097	0.106	0.106												
6 *O.convictus* B628	0.118	0.118	0.125	0.125	0.041											
7 *O.convictus* B629	0.095	0.095	0.101	0.101	0.006	0.038										
8 *O.makazayazaya* GP21	0.102	0.102	0.111	0.111	0.111	0.110	0.105									
9 *O.makazayazaya* KIZ02143	0.098	0.098	0.115	0.115	0.117	0.115	0.111	0.007								
10 *O.makazayazaya* YPX53011	0.100	0.104	0.128	0.128	0.113	0.099	0.107	0.038	0.037							
11 *O.malhotrae* GP2041	0.102	0.102	0.108	0.108	0.092	0.094	0.086	0.081	0.083	0.084						
12 *O.malhotrae* GP2053	0.102	0.102	0.108	0.108	0.092	0.094	0.086	0.081	0.083	0.084	0.000					
13 *O.malhotrae* ROM 39381	0.110	0.110	0.104	0.104	0.095	0.097	0.090	0.079	0.084	0.086	0.007	0.007				
14 *O.tonkinensis* GP1665	0.105	0.101	0.119	0.119	0.096	0.096	0.094	0.095	0.100	0.109	0.081	0.081	0.083			
15 *O.tonkinensis* KIZ011602	0.107	0.103	0.120	0.120	0.098	0.102	0.099	0.098	0.103	0.113	0.084	0.084	0.086	0.011		
16 *O.zayuensis* GP713	0.085	0.085	0.110	0.110	0.090	0.092	0.088	0.085	0.085	0.094	0.060	0.060	0.062	0.089	0.088	
17 *O.zayuensis* GP1505	0.088	0.088	0.106	0.106	0.092	0.094	0.090	0.087	0.090	0.095	0.065	0.065	0.067	0.094	0.094	0.019

### ﻿Taxonomic account

#### ﻿Viperidae Oppel, 1811

##### ﻿ Ovophis jenkinsi sp. nov.

Taxon classificationAnimaliaSerpentesViperidae

0C2A2191-3B58-51BB-A4F5-1908B7D89EE7

https://zoobank.org/45FF4F16-3F01-4ADC-8DA7-24E84E3B810D

###### Type material.

**Figure 2.**
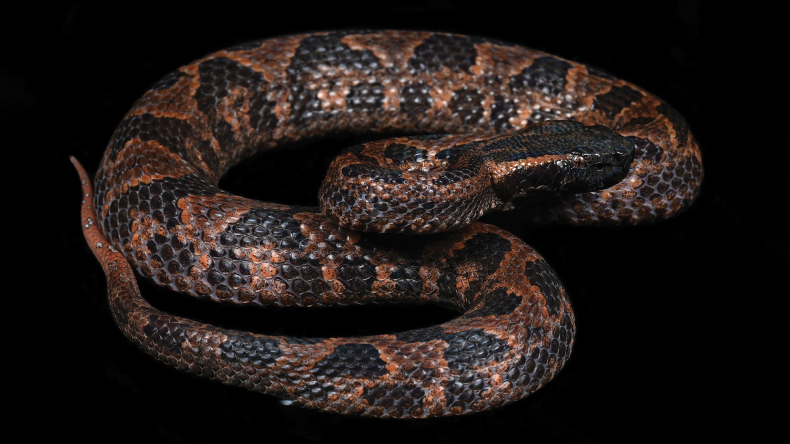
Holotype of *Ovophisjenkinsi* sp. nov. (IOZ 002679) in life.

**Figure 3.**
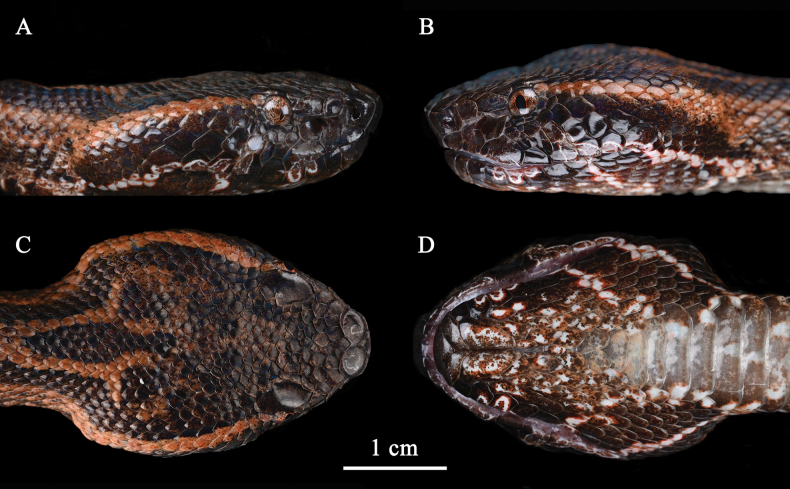
Head of the holotype of *Ovophisjenkinsi* sp. nov. (IOZ 002679) **A** lateral (right) view **B** lateral (left) view **C** dorsal view **D** ventral view.

**Figure 4.**
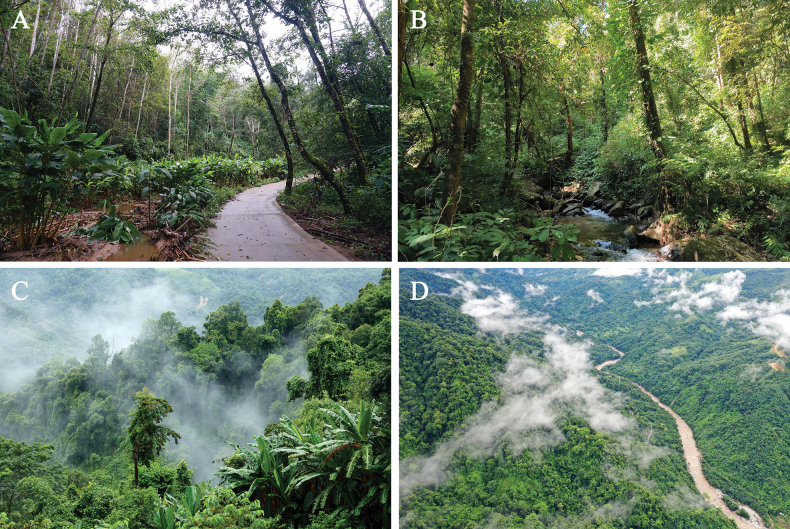
Habitat of *Ovophisjenkinsi* sp. nov. at the type locality in Tongbiguan Township, Yingjiang County, Yunnan Province, China **A** microhabitat, photographed by Sheng-Chao Shi **B** microhabitat, photographed by Guo-Wei Mo **C, D** macrohabitats, photographed by Xiao-Jun Gu.

***Holotype*.**IOZ 002679, an adult male (Figs 2, 3) from Tongbiguan Township, Yingjiang County, Yunnan Province, China (24°36′33″N, 97°39′29″E; 1,343 m a.s.l.) (Fig. 4). It was collected near the road by Zhong-Wen Jiang and Xian-Chun Qiu.

***Paratype*.**IOZ 002680 and YJ201801, adult females from Tongbiguan Township, Yingjiang County, Yunnan Province, China (24°35′04″N, 97°41′13″E; 1,321 m a.s.l.) collected by Zhong-Wen Jiang and Xian-Chun Qiu in October 2023 and 2018; juveniles YJ201802 and YJ201803 from the same locality collected by Zhong-Wen Jiang in October 2018.

###### Etymology.

The specific epithet of the new species is dedicated to Robert “Hank” William Garfield Jenkins AM (August 1947–September 2023), a herpetologist and former chairman of the CITES Animals Committee from Australia, with a passion for snakes, especially pitvipers, and helped China, along with many Asian countries, complete snake census, conservation, and management projects. We suggest the common name “Jenkins’ mountain pitviper” in English and “yíng jiāng lào tiě tóu shé” (盈江烙铁头蛇) in Chinese.

###### Diagnosis.

*Ovophisjenkinsi* sp. nov. can be distinguished by the following combination of morphological characters: (1) internasals in contact or separated by one small scale; (2) second supralabial entire and bordering the loreal pit; (3) dorsal scales in 23 (25)–21 (23, 25)–19 (17, 21) rows; (4) 134–142 ventrals; (5) 40–52 pairs of subcaudals; (6) third supralabial larger than fourth in all examined specimens of *Ovophisjenkinsi* sp. nov.; (7) deep orange-brown or dark brownish-grey markings on dorsal head surface; (8) background color of dorsal surface deep orange-brown or dark brownish-grey; (9) both sides of dorsum display dark brown trapezoidal patches; (10) scattered small white spots on dorsal surface of tail.

###### Description of holotype.

Adult male; body stout and robust, medium-sized, tail slender, TL 515.9 mm (SVL 421.0 mm, TAL 94.9 mm, TAL/TL: 0.23); head triangular in dorsal view, moderately distinct from neck, longer than width, HL 26.6 mm, HW 18.6 mm (HW/HL: 0.70). Snout blunt and rounded, rostral trapezoidal, broader than high, RW 4.6 mm, RH 3.5 mm (RW/RH: 1.31; RW/HW: 0.25), upper edge visible from dorsum; eye small, ED 2.7 mm (ED/HL 0.10), pupil vertical; nostril subcircular, located in the middle of nasal; nasal divided into two scales by nostril; two internasals, elliptical, separated anteriorly by a small scale and bordered by the upper edge of rostral, connected posteriorly; loreal single; two preoculars, in contact with eye posteriorly; two postoculars, upper one in contact with the lower edge of supraocular; subocular single and elongate, respectively separated by two small scales from the third, fourth and fifth supralabials; supraocular single, the largest scales on the dorsal surface of head, separated by 7–8 scales; supralabials eight, first and second in contact with nasal, second entire and bordering the loreal pit, third larger than fourth; 11 infralabials on left (seventh and eighth infralabials bipartitioned relative to right), 10 infralabials on right, first pair in broad contact with each other, first to third in contact with chin shields; mental triangular; one pair of chin shields, meeting in midline, the right one slightly larger than the left; dorsal scales in 25–21–19 rows, bluntly keeled, except outer row; 134 ventrals, excluding six preventrals; subcaudal scales 49, paired, excluding tail tip; cloacal plate entire.

###### Coloration in life.

Dorsal head surface black, with deep-orange blotches; a deep orange marking resembling an open pair of surgical scissors exists on the front of neck; a deep-orange stripe exists from the upper postocular to the anterior nape, the stripe demarcated from black dorsal head at top, gradually transitioning to black at bottom, approximately one scale row in width behind orbit of eye, after three scales, approximately two scales rows in width, enlarge to 3–4 scales rows in width on the posterior of head. Lateral head surface black, tiny white and vermilion spots exist on the surface of scales near snout; an irregular stripe extends from subocular to the fifth and sixth infralabials, the outermost ring of vermilion, subtle, second ring of white, obvious; the stripe splits in two at fifth and sixth supralabials, one extending backward through seventh, eighth supralabials and the last two infralabials, the other extending downward through seventh and eighth infralabials (left) and seventh infralabial (right), converging at the outer row of dorsal scales; similar markings exist on the third supralabial and third to fifth infralabials. Background color of ventral head surface deep orange, mixed with irregular white blotches with vermilion edges. Pupil black; iris deep orange mixed with white and black.

Background color of dorsal surface deep orange, with 18 connected or disconnected dark brown patches on both sides of body and three similar spots on anterior section of tail visible from dorsum; dorsal blotches predominantly trapezoidal, approximately 2–6 scales in length, and 4–5 scales rows in width, mottled with a few deep orange tiny spots on most dark brown patches; two clusters of lateral dark brown patches exist under each dorsal dark brown patch, each patch covers 2–3 dorsal scales and separated from ventral scales by 2–3 rows of dorsolateral scales. Posterior section of tail pink, 21 tiny spots exist on the dorsal surface, spots white with brown edges, no more than a scale in size. Mixed cream and tan on ventral surface of anterior tail and body, clean pink on posterior section of tail.

###### Intraspecific variation.

Morphometric data are summarized in Table 4. Dorsal head surface of each paratype specimen has different approximately symmetrical markings respectively. Internasals are separated by one scale, and the dorsal background color is dark brownish-grey in paratypes IOZ 002680 and YJ201801. Light greyish-brown on background color of dorsal body in juveniles YJ201802 and YJ201803. Third to tenth subcaudals unpaired in YJ201803. The patches on dorsal body are mostly rectangular in IOZ 002680.

**Table 4.** Scalation data and measurements (in mm) of *Ovophisjenkinsi* sp. nov.

	IOZ 002679 Holotype	IOZ 002680 Paratype	YJ201801 Paratype	YJ201802 Paratype	YJ201803 Paratype
Sex	Male	Female	Female	Juvenile	Juvenile
TL	515.9	402.3	690.0	261.0	279.0
TAL	94.9	66.9	91.1	43.8	45.0
HL	26.6	26.0	38.3	16.2	16.8
HW	18.6	20.6	28.8	11.7	11.2
HW/HL	0.70	0.79	0.75	0.72	0.67
PRO	2	2	2	2	2
SBO	1	3	2	2	2
PO	2	3	2	2	3
SL	8/8	9/9	8/10	8/–	8/8
IL	11/10	12/12	11/11	11/11	11/11
DSR	25–21–19	25–23–19	25–25–21	23–21–19	23–23–17
VS	134	142	138	134	135
SC	49	40	40	50	52

Note: the missing data are marked as “–”.

###### Comparisons.

*Ovophisjenkinsi* sp. nov. can be distinct from other congeneric species by the following characters (Table 5): internasals in contact or separated by one small scale (vs internasals separated by two small scales in *O.malhotrae*); second supralabial entire and bordering the loreal pit (vs second supralabial bordering the loreal pit or separated by a loreal in *O.makazayazaya*, *O.tonkinensis*, and *O.zayuensis*); dorsal scales in 23 (25)–21 (23, 25)–19 (17, 21) rows (vs dorsal scales 23 (21)–23 (21)–19 in *O.monticola*, 25 (27, 29)–23 (21–25)–19 (21) in *O.tonkinensis*, 27–23–19 in *O.malhotrae* and 25 (27)–23–19 (17) in *O.zayuensis*); 134–142 ventrals (vs 141–172 ventrals in *O.monticola*, 145 in *O.malhotrae* and 160–177 in *O.zayuensis*); subcaudal scales in pairs (vs unpaired in *O.tonkinensis* and *O.zayuensis*); 40–52 pairs of subcaudals (vs 17–31 pairs in *O.convictus*); the third supralabial being larger than fourth (vs fourth larger than third supralabial in *O.makazayazaya* and *O.tonkinensis*); deep orange-brown or dark brownish-grey markings on dorsal head surface (vs no markings on dorsal head surface in *O.convictus* and *O.tonkinensis*); background color of dorsal surface deep orange-brown or dark brownish-grey (vs yellowish-brown or dark-grey in *O.makazayazaya*, yellowish-brown in *O.monticola* and *O.tonkinensis*, reddish-brown or brown in *O.zayuensis*); both sides of dorsum display dark brown trapezoidal patches (vs mostly rectangular patches in *O.monticola*); adults HW/HL 0.70–0.79 (vs 0.64–0.65 in *O.monticola*); scattered small white spots on dorsal surface of tail (vs continuous small white spots on dorsal surface of tail in *O.tonkinensis* and *O.malhotrae*).

**Table 5.** Morphological comparison of *Ovophis* species.

	DSR	VS	SC	Does the 2^nd^SL border the loreal pit	3^rd^ and 4^th^SL	Dosal head surface	Dorsal background color	Dorsal patches	White spots on dorsal surface of tail
*Ovophisjenkinsi* sp. nov.	23 (25)–21 (23, 25)–19 (17, 21)	134–142	40–52, paired	Yes	3^rd^ > 4^th^	Patterned	Deep orange-brown or dark brownish-grey	Mostly trapezoidal	Scattered
*O.monticola*	23 (21)–23 (21)–19	141–172	37–58, paired	Yes	3^rd^ > 4^th^	Patterned	Yellowish-brown	Mostly rectangular	Scattered
*O.convictus*	25–25–18	136	17–31, paired	Yes	3^rd^ > 4^th^	Unpatterned	Yellowish-brown	Mostly rectangular	Scattered
*O.makazayazaya*	25 (27, 29)–23 (25, 21)–19 (21)	131–159	34–52, paired	Yes or no	3^rd^ < 4^th^	Patterned or unpatterned	Yellowish-brown or dark-grey	Rectangular or irregular patches	Scattered
*O.malhotrae*	27–23–19	145	47, paired	Yes	3^rd^ > 4^th^	Patterned	Dark-brown	Mostly rectangular	Continuous
*O.tonkinensis*	25 (27, 29)–23 (21–25)–19 (21)	128–134	39–49, unpaired	Yes or no	3^rd^ < 4^th^	Unpatterned	Yellowish-brown	Rectangular or irregular patches	Continuous
*O.zayuensis*	25 (27)–23–19 (17)	160–177	43–64, unpaired	Yes or no	3^rd^ > 4^th^	Patterned or unpatterned	Reddish-brown or brown	Mostly trapezoidal and triangular	No visible white spots

###### Distribution and ecology.

**Figure 5.**
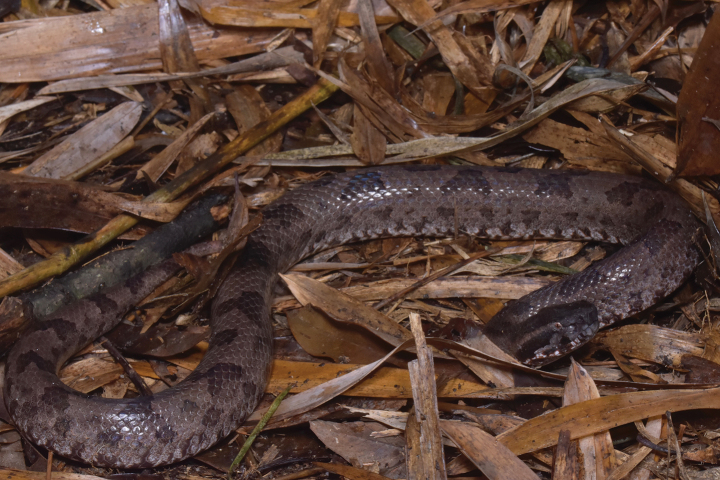
*Ovophisjenkinsi* sp. nov. and its microhabitat. Photographed by Zhong-Wen Jiang in Yingjiang, Yunnan.

*Ovophisjenkinsi* sp. nov. is currently known only from Yingjiang County, Yunnan Province, China. It was found in the tropical montane rainforest at an altitude of around 1,300 m. Overlapping herpetofauna includes *Lycodonchapaensis* (Angel & Bourret, 1933), *Trimeresuruspopeiorum* Smith, 1937, *Pseudocalotesjingpo*Xu et al., 2024, and other species (Xu et al. 2024). The new species reaches activity peak in autumn and is active nocturnally during light rain or high humidity, at temperatures around 15–22 °C (Fig. 5). The type specimens were collected at night in October. When threatened, these snakes inflate their bodies to make themselves appear larger and strike quickly. The specimen IOZ 002680 had released odour from the cloacal scent glands when captured. We are currently unsure of the feeding habit of *O.jenkinsi* sp. nov. in the wild. They fed on juvenile mice (*Musmusculus* Linnaeus, 1758) in our captivity observations. Therefore, we presume the species prey on small mammals in the wild.

## ﻿Discussion

The phylogenetic topology in this study supports Zeng et al. (2023): *Ovophismalhotrae* sistering *O.zayuensis* (Zeng et al. 2023). However, the phylogenetic topology in this study differs from previous publications (Malhotra et al. 2011; Zeng et al. 2023). In this study, *O.tonkinensis* clustered with all other congeners (excluding “*O.*” *okinavensis*), while it clustered with *O.makazayazaya* in Malhotra et al. (2011) and Zeng et al. (2023). Thus, introducing a larger quantity of genetic dataset is suggested when conducting further phylogenetic studies of genus *Ovophis*.

In recent years, new snake species have been discovered constantly near the Yunnan border (Jiang et al. 2020; Chen et al. 2021; Hou et al. 2021; Lee 2021; Liu et al. 2021; Shi et al. 2022; Ma et al. 2023). Zeng et al. (2023) described the new species *O.malhotrae* based on specimens from southern Yunnan, and its molecular systematic position indicated that several populations from Vietnam and Laos may refer to cryptic species of the genus *Ovophis*. Thus, snake diversity in this region may have been underestimated in previous studies.

**Figure 6.**
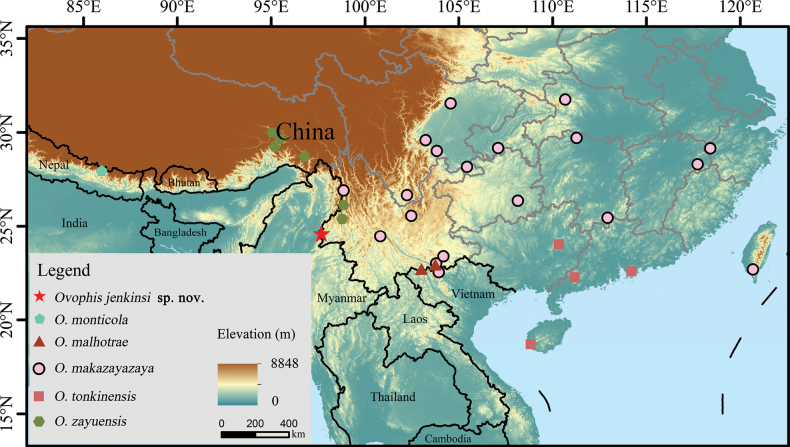
The type locality of *Ovophisjenkinsi* sp. nov. and some distribution sites of *Ovophis* species in China.

According to field surveys and recent publications, we identified updated distribution sites of *Ovophis* species in China (Zhang et al. 2011; Che et al. 2020; Zeng et al. 2023; Uetz et al. 2024) (Fig. 6). In Yunnan, *O.makazayazaya* is widely distributed in most parts of the province, *O.zayuensis* in the Gaoligong Mountain region of western Yunnan, and *O.malhotrae* in Jinping and Pingbian in southeastern Yunnan (Wang et al. 2022; Zeng et al. 2023). In some areas, there may be overlapping distributions of *O.makazayazaya* and other congeners. The specimens of “*Trimersurusmonticola*” from Hotha, Longchuan County, Yunnan Province, cited by Anderson (1878a), displayed intra-specific polymorphism: the second supralabial completed or divided from the anterior of loreal pit, consistent with the character of *O.makazayazaya* (Guo et al. 2021). Further examinations through photos of the specimen that was recorded by Anderson (1878b) were conducted and showed that it displays 10 supralabials, the second divided from the loreal pit and fourth larger than third, suggesting taxonomic placement under *O.makazayazaya*. However, since Longchuan County is adjacent to Yingjiang County, and in specimens from Hotha, Longchuan, the second supralabial also reaches the loreal pit, they should belong to *O.jenkinsi* sp. nov. In addition, Yingjiang County, Yunnan Province is located on the border of China and is adjacent to Myanmar; thus, the new species may also be distributed in Myanmar.

The new species is morphologically most similar to *O.monticola*, but can be distinguished by morphological characters such as wider head, fewer ventrals, trapezoidal patches on dorsal body, and deep orange-brown or dark brownish-grey dorsal surface rather than yellowish brown. In the specimens examined, the new species has a maximum TL of 690 mm (specimen YJ201801), while *O.monticola* appears to be larger, with a maximum TL of 1,300 mm (Sharma et al. 2013). We will collect more specimens of *O.jenkinsi* sp. nov. in the future to supplement the morphological data. Molecular phylogenetic analyses show *O.jenkinsi* sp. nov. is genetically differentiated from *O.malhotrae* (*p*-distance 10.2–11.0). Currently, only the holotype of *O.malhotrae* is described in supporting documents, the intraspecific variation of this species is unclear. It is, therefore, suggested that additional samples from along the Yunnan border and adjacent areas would enrich the current morphological dataset of *O.jenkinsi* sp. nov., *O.malhotrae*, and other *Ovophis* species and support further biodiversity discoveries.

## Supplementary Material

XML Treatment for Ovophis jenkinsi

## ﻿Additional information

### Author ORCIDs

Xian-Chun Qiu https://orcid.org/0009-0001-5776-711X

Jing-Song Shi https://orcid.org/0000-0001-9168-1734

### Data availability

All of the data that support the findings of this study are available in the main text.
